# Unravelling Recombination Processes in Bifacial Guanidinium-Incorporated Perovskite Solar Cells with SnO_2_ and TiO_2_ ETLs

**DOI:** 10.3390/ma19112374

**Published:** 2026-06-03

**Authors:** Hryhorii Parkhomenko, Adem Karakuzu, Sanjay Sahare, Mykhailo Solovan, Marcin Ziółek

**Affiliations:** Faculty of Physics and Astronomy, Adam Mickiewicz University, Uniwersytetu Poznańskiego 2, 61-614 Poznań, Poland; hryhorii.parkhomenko@gmail.com (H.P.); adekar@st.amu.edu.pl (A.K.); sanjay.sahare@amu.edu.pl (S.S.)

**Keywords:** bifacial solar cells, perovskite, charge recombination, guanidinium

## Abstract

Maximising the energy yield of perovskite solar cells (PSCs) through bifacial architectures is a promising route toward commercialisation. However, optimising charge extraction at the interfaces remains a critical challenge. In this study, we systematically compare tin dioxide (SnO_2_) and titanium dioxide (TiO_2_) electron transport layers (ETLs) in bifacial guanidinium-incorporated PSCs with a transparent gold (10 nm) back electrode. While the bulk perovskite crystallinity remains invariant on both substrates, SnO_2_ provides a distinct optical advantage through enhanced UV-blue transmittance. Beyond these optical benefits, comprehensive recombination process analyses reveal that SnO_2_ drastically suppresses non-radiative recombination. The SnO_2_ layer effectively mitigates defect states, significantly reducing both bulk and surface trap-assisted recombination rates without disrupting intrinsic bimolecular charge transport. Ultimately, these findings underscore the critical importance of rational interfacial engineering to neutralise defects, proving SnO_2_ to be an indispensable component for realising highly efficient and commercially viable bifacial perovskite optoelectronics.

## 1. Introduction

Organic–inorganic halide perovskite solar cells (PSCs) have experienced an unprecedented trajectory in power conversion efficiency (PCE), positioning themselves as highly promising candidates for next-generation photovoltaics [1,2,3,4,5,6,7]. As the field shifts its focus from purely champion efficiencies toward practical commercialisation, maximising the energy yield per unit area has become a critical objective. In this context, bifacial PSCs have garnered significant attention. By harvesting albedo and scattered light from the rear side in addition to direct front-side illumination, bifacial architectures can substantially boost the overall power output without requiring an increase in the device footprint [8,9,10,11,12,13].

Despite these advancements, the commercial viability of 3D perovskites is frequently hindered by their inherent vulnerability to moisture, heat, and light [14,15,16,17,18]. Consequently, the incorporation of large organic spacer cations, such as guanidinium (GA), into the perovskite lattice has emerged as a robust alternative to significantly enhance structural and environmental stability [19,20,21]. However, the introduction of these bulky cations can impede out-of-plane charge transport and introduce complex interfacial dynamics, making the optimisation of charge extraction layers paramount, especially in bifacial designs where charge carriers are generated across varying depths depending on the illumination direction.

The electron transport layer (ETL) plays a pivotal role in dictating the charge extraction efficiency and minimising interfacial recombination in PSCs. Traditionally, titanium dioxide (TiO_2_) has been widely used as an ETL. However, it suffers from relatively low electron mobility and a high density of surface trap states, which can exacerbate non-radiative recombination and hysteresis [22,23,24]. Tin dioxide (SnO_2_) has recently emerged as a highly attractive alternative due to its deeper conduction band, superior electron mobility, excellent UV stability, and processing compatibility at lower temperatures [25,26,27]. While the advantages of SnO_2_ have been widely reported in standard monolithic architectures, a rigorous comparative analysis of ETL dynamics specifically within the unique optoelectronic environment of bifacial, GA-based PSCs remains underexplored.

In this study, we present a comprehensive comparative analysis of SnO_2_ and TiO_2_ as ETLs in bifacial GA-based perovskite solar cells, utilising an ultrathin, transparent gold (10 nm) back electrode. We coupled steady-state photovoltaic evaluation with advanced transient techniques, including light intensity-dependent measurements and open-circuit voltage decay (OCVD). Furthermore, we quantitatively extracted the specific recombination rate constants to isolate bulk and surface trap-assisted processes from intrinsic bimolecular recombination. Our findings demonstrate that while the fundamental bulk crystallinity of the perovskite remains invariant between the substrates, the SnO_2_ ETL architecture delivers superior bifacial performance, achieving a bifaciality factor of 0.74. The modelling definitively proves that this enhancement is driven by a nearly an order-of-magnitude reduction in both bulk and interfacial non-radiative recombination.

## 2. Materials and Methods

### 2.1. Materials

Fluorine-doped tin oxide (FTO)-coated glass substrates with a sheet resistance of 7–8 Ω/sq were supplied by OPVtech (Yingcou, China). An aqueous colloidal dispersion of tin dioxide nanoparticles (SnO_2_ NPs, 15 wt%) was procured from Alfa Aesar (Ward Hill, MA, USA). 30NR-D titania paste (average nanoparticle size 30 nm) and methylammonium chloride (MACl) were sourced from Greatcell Solar Materials (Queanbeyan, Australia). Lead (II) iodide (PbI_2_), guanidinium iodide (GAI), methylammonium iodide (MAI), titanium diisopropoxide bis(acetylacetonate), Spiro-MeOTAD, lithium bis(trifluoromethanesulfonyl)imide (LiTFSI), tert-butylpyridine (t-BP), anhydrous N,N-dimethylformamide (DMF), dimethyl sulfoxide (DMSO), ethyl acetate, chlorobenzene, and acetonitrile were purchased from Merck/Sigma-Aldrich (Darmstadt, Germany).

### 2.2. Device Fabrication

Perovskite solar cells with a n-i-p architecture were fabricated on FTO glass substrates (2.5 cm × 2.5 cm). The substrates were subjected to a rigorous cleaning protocol consisting of sequential 15 min ultrasonic baths in detergent, deionised water, acetone, and 2-propanol. After drying with a stream of air, a 30 min UV–ozone treatment was applied to eliminate residual organics and improve wettability.

Two distinct device configurations were fabricated to compare the electron transport layers. The SnO_2_-based architecture consisted of Glass/FTO/SnO_2_/Perovskite/Spiro-MeOTAD/Au. The TiO_2_-based devices were structured as Glass/FTO/c-TiO_2_/m-TiO_2_/Perovskite/Spiro-MeOTAD/Au.

For the SnO_2_ ETL, the commercial NPs dispersion was diluted in deionised water (1:4 *v*/*v*), spin-cast onto the FTO at 4000 rpm for 30 s, and subsequently thermally annealed at 150 °C for 30 min. Conversely, the TiO_2_ ETL comprised a bilayer system. The compact TiO_2_ (c-TiO_2_) layer was deposited via spray pyrolysis at 450 °C utilising a titanium diisopropoxide precursor solution (1 mL diluted in 14 mL of ethanol). A mesoporous TiO_2_ (m-TiO_2_) layer was then deposited via spin-coating (2000 rpm, 10 s) using ethanol-diluted titania paste (1:6 *w*/*w*) in ambient air, followed by a 30 min calcination step at 500 °C.

Perovskite film deposition was carried out in a nitrogen-filled glovebox. The GA(MA)_5_Pb_5_I_16_ active layer was prepared as a 1.5 M precursor solution (PbI_2_ (691.25 mg), MAI (238.4 mg), and GAI (56 mg) in a 1:5:5 molar ratio within a 1 mL solvent blend of DMF and DMSO (4:1 *v*/*v*)). MACl was introduced as an additive at a concentration of 12.5 mg/mL. The perovskite solutions were spun at 4000 rpm for 20 s, with 250 µL of ethyl acetate dripped onto the centre of the spinning substrate 10 s prior to the program’s completion. The resulting films underwent crystallisation via thermal annealing at 150 °C for 20 min.

To form the hole transport layer (HTL), a chlorobenzene solution containing 72 mg/mL of Spiro-MeOTAD was prepared and doped with 29 µL of t-BP and 17.5 µL of a primary Li-TFSI stock solution (520 mg Li-TFSI per 1 mL acetonitrile). The HTL was spin-coated at 4000 rpm for 30 s, and the films were aged overnight in a dry air environment. Finally, a 10 nm top gold electrode was deposited via DC sputtering.

### 2.3. Material and Device Characterisation

Crystallographic analysis of the perovskite layers was conducted using a Rigaku (Tokyo, Japan) SmartLab X-ray diffractometer equipped with a nickel-filtered Cu Kα radiation source (λ = 1.5418 Å). Optical absorption profiles were acquired utilising a JASCO V-770 UV-VIS-NIR spectrophotometer (Tokyo, Japan). Photovoltaic performance (*J-V* curves) and impedance spectroscopy were evaluated using an Autolab M101 potentiostat (Herisau, Switzerland) integrated with an Instytut Fotonowy solar simulator (Krakow, Poland). The system provided standard AM 1.5G, 1 Sun illumination, calibrated against an ABET 15151 silicon reference cell (Milford, CT, USA). Frequency-dependent impedance data (10 Hz to 10 MHz) were collected in both dark and 1 Sun conditions, applying a 40 mV AC perturbation and sweeping from a negative bias up to the open-circuit voltage (*V_oc_*). Transient *V_oc_* decay dynamics were measured using a high-speed white LED light source (100 mW/cm^2^) governed by an SRS DG645 digital delay generator (Sunnyvale, CA, USA). Signals were recorded on a Tektronix DPO 4104B-L digital phosphor oscilloscope (Beaverton, OR, USA) interfaced with a 1 GΩ high-impedance buffer (200 MHz bandwidth). These measurements were conducted inside a grounded Faraday cage to minimise electromagnetic interference. Femtosecond transient absorption (TA) spectroscopy measurements were conducted directly on the fabricated cells using the setup described earlier [28,29]. The samples were excited using a 440 nm pump pulse with an energy of 20–50 nJ to selectively probe the regions adjacent to the interfaces. The resulting kinetics were monitored via the bleach signal peaking at 760 nm.

## 3. Results

**Figure 1:**
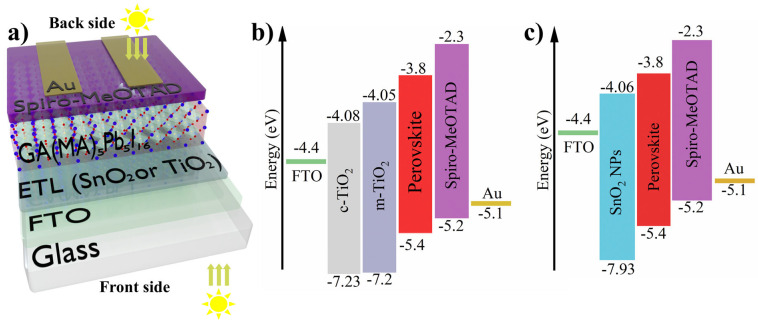
(**a**) Schematic illustration of the bifacial perovskite solar cell architecture (Glass/FTO/ETL/Perovskite/Spiro-MeOTAD/Au). Energy-level diagrams illustrating the band alignments for devices utilising (**b**) TiO_2_ and (**c**) SnO_2_ as the electron transport layer.

Figure 1a illustrates the three-dimensional schematic of the device architecture, which follows an n-i-p configuration: Glass/FTO/ETL (SnO_2_ or TiO_2_)/Perovskite (GA(MA)_5_Pb_5_I_16_)/Spiro-MeOTAD/Au. The photoactive layer consists of a GA-incorporated perovskite, positioned between the ETL and the Spiro-MeOTAD hole transport layer. A 10 nm thin gold (Au) layer is utilised as the back contact, engineered to allow light penetration for bifacial illumination. The energy-level diagrams based on the work functions of the material [30,31,32,33] for devices employing TiO_2_ and SnO_2_ ETLs are presented in Figure 1b and Figure 1c, respectively.

As shown in Figure S1a, the X-ray diffraction (XRD) pattern of the GA-incorporated perovskite film exhibits dominant, highly crystalline peaks at approximately 14° and 28°, corresponding to the (110) and (220) crystallographic planes, in agreement with the previous reports for the same perovskite [21]. Furthermore, steady-state UV-Vis absorbance spectroscopy (Figure S1b) reveals that the absorption profiles of the perovskite layers deposited on SnO_2_ and TiO_2_ are nearly identical, both exhibiting a sharp absorption onset around 750–780 nm.

While the bulk perovskite absorption remains consistent, the optical properties of the front contact stack itself differ significantly. Figure S2 compares the optical transmittance of the bare substrates (Glass/FTO/SnO_2_ and Glass/FTO/TiO_2_). The SnO_2_-coated substrate demonstrates notably higher transmittance across the visible spectrum, particularly in the lower wavelength region (300–450 nm), whereas the TiO_2_ layer exhibits significant parasitic absorption in this UV-blue region due to its bandgap characteristics. This optical transparency of the SnO_2_ ETL ensures that a larger fraction of incident photons successfully reaches the perovskite absorber layer, establishing a baseline optical advantage for charge generation under front-side illumination.

### 3.1. Photovoltaic Performance and Bifaciality

**Figure 2:**
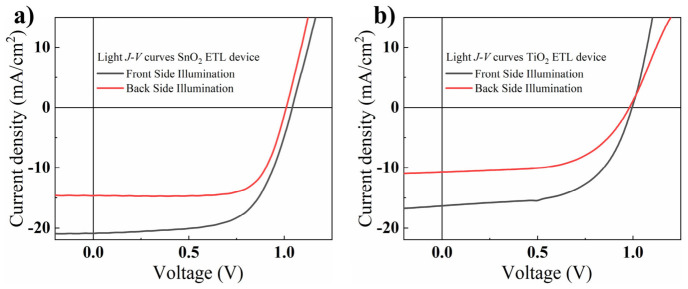
*J-V* curves of the bifacial perovskite solar cells with (**a**) SnO_2_ and (**b**) TiO_2_ as the electron transport layer, measured under AM 1.5G front- and back-side illumination.

To quantitatively assess the photovoltaic performance of the bifacial GA-incorporated perovskite solar cells, current density–voltage (*J-V*) measurements were conducted under standard AM 1.5G (100 mW/cm^2^) illumination. The cells were evaluated under both front-side (illumination through the glass/FTO) and back-side (illumination through the transparent Au back electrode) conditions. The corresponding *J-V* curves are presented in Figure 2, and the extracted photovoltaic parameters, *V_oc_*, short-circuit current density (*J_sc_*), fill factor (*FF*), and *PCE*, are summarised in Table 1.

The devices with the SnO_2_ ETL demonstrated superior photovoltaic parameters across all illumination configurations compared to the TiO_2_. Under standard front-side illumination, the SnO_2_-based device achieved a *PCE* of 13.8%, driven by a robust *J_sc_* of 20.9 mA/cm^2^, a *V_oc_* of 1.03 V, and an *FF* of 0.64. The significantly elevated *J_sc_* in the SnO_2_ architecture is directly attributable to its superior optical transmittance in the UV-blue region, which minimises parasitic absorption and maximises the photon flux reaching the perovskite absorber. In contrast, under front-side illumination, the TiO_2_ device yielded a *PCE* of only 9.7%, suffering from a lower *J_sc_* of 16.7 mA/cm^2^, a *V_oc_* of 0.99 V, and a reduced *FF* of 0.59.

When illuminated from the back side, both device architectures predictably exhibited a reduction in efficiency. The primary cause of this performance reduction is the expected optical loss (parasitic absorption and reflection) from the gold and Spiro-MeOTAD layers, compounded by a shifted carrier generation profile. However, the SnO_2_ device maintained robust performance, yielding a PCE of 10.2% (*J_sc_* = 14.7 mA/cm^2^, *V_oc_* = 1.01 V) and an improved fill factor (*FF* = 0.69) compared to its front illumination. Conversely, the limitations of the TiO_2_ layer are further exacerbated under back-side illumination, where the efficiency drops to 6.2%, accompanied by a severe reduction in photocurrent (*J_sc_* = 10.9 mA/cm^2^) and a fill factor (*FF* = 0.58).

The overarching advantage of the SnO_2_ transport layer is most clearly reflected in the bifaciality factor, defined as the ratio of back-side *PCE* to front-side *PCE*. The SnO_2_ devices achieved a highly competitive bifaciality factor of 0.74, outperforming the TiO_2_ devices, which managed a factor of only 0.64. This robust bifacial performance signifies that the SnO_2_ architecture not only facilitates highly efficient extraction for charge carriers generated locally near the ETL but also effectively extracts carriers generated deep within the active layer during back-side illumination.

To further evaluate the inherent electrical properties and charge transport behaviour of the devices in the absence of photogenerated carriers, dark *J-V* characteristics were measured (Figure S3a). The built-in potential (*V_bi_*), which provides the fundamental thermodynamic driving force for the separation and extraction of charge carriers, can be extracted from the onset of the exponential forward bias injection current [34,35]. The SnO_2_-based device exhibits a slightly higher *V_bi_* of 0.93 V compared to 0.92 V for the TiO_2_-based device. This higher built-in potential in the SnO_2_ architecture facilitates a stronger internal electric field, contributing to more efficient charge extraction and supporting the higher *V_oc_* observed under illumination [36,37].

Furthermore, the voltage-dependent differential resistance (*R_diff_*) was derived from the dark *J-V* curves to analyse the parasitic resistances of the cells (Figure S3b). At low forward bias, both devices exhibit exceptionally high shunt resistance (*R_sh_* > 10^6^ Ω), indicating excellent film coverage with minimal pinholes and low leakage current pathways for both ETLs. As the forward bias increases beyond the built-in potential, the differential resistance drops sharply, converging toward the series resistance (*R_s_*) of the devices. The low *R_s_* values for both configurations confirm good ohmic contact at the interfaces.

### 3.2. Recombination Dynamics

Next, we investigated the dependence of the *V_oc_* and *J_sc_* on the incident light intensity (*I*), to gain deeper insights into the charge carrier recombination kinetics and understand the origin of the performance differences between the two ETLs.

The relationship between *V_oc_* and light intensity provides critical information regarding the dominant recombination mechanisms operating within the devices at *V_oc_* [38,39,40,41]. This relationship is typically expressed as *V_oc_* ∝ *nln*(*I*)(*kT*/*q*), where *k* is the Boltzmann constant, *T* is the absolute temperature, *q* is the elementary charge, and *n* represents the diode ideality factor. An ideality factor close to 1 indicates that bimolecular (band-to-band) recombination dominates, whereas a value approaching 2 signifies that trap-assisted, Shockley–Read–Hall (SRH) non-radiative recombination is the primary loss mechanism.

**Figure 3:**
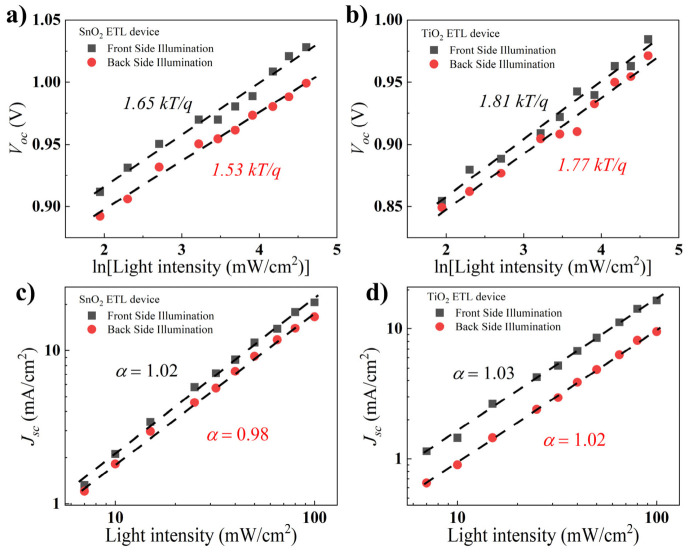
Light intensity-dependent *V_oc_* (**a**,**b**) and *J_sc_* (**c**,**d**) measurements of the bifacial devices with SnO_2_ and TiO_2_ ETLs under front and back illumination.

As shown in Figure 3a,b, the *V_oc_* versus logarithmic light intensity plots were extracted for both devices under front and back illumination. The SnO_2_-based devices exhibited ideality factors of 1.65 under front illumination and 1.53 under back illumination. In contrast, the TiO_2_-based devices displayed noticeably higher slopes of 1.81 and 1.77 for front and back illumination, respectively. The significantly lower ideality factors observed in the SnO_2_ devices confirm a substantial reduction in trap-assisted non-radiative recombination [38].

Furthermore, the dependence of *J_sc_* on light intensity was evaluated to assess charge transport and extraction efficiency under short-circuit conditions [42,43,44]. This relationship follows a power law, *J_sc_* ∝ *I^α^*, where an α value close to 1 suggests that bimolecular recombination is negligible and charge carriers are swept out of the device efficiently prior to recombining. As depicted in Figure 3c,d, the fitted *α* values for the SnO_2_ devices are 1.02 (front) and 0.98 (back), while the TiO_2_ devices exhibit values of 1.03 (front) and 1.02 (back). Since all *α* values are nearly unity, it is evident that space–charge-limited currents and bimolecular recombination losses at short-circuit conditions are minimal [45,46] for both ETL configurations, regardless of the illumination direction.

To further elucidate the charge carrier recombination kinetics and directly quantify the carrier lifetimes under operational conditions, transient OCVD measurements were performed. By monitoring the decay of *V_oc_* in the dark immediately after switching off the illumination, we can decouple the charge extraction processes from recombination, allowing for a direct assessment of the non-radiative recombination losses [47,48], particularly at the perovskite/ETL interface.

**Figure 4:**
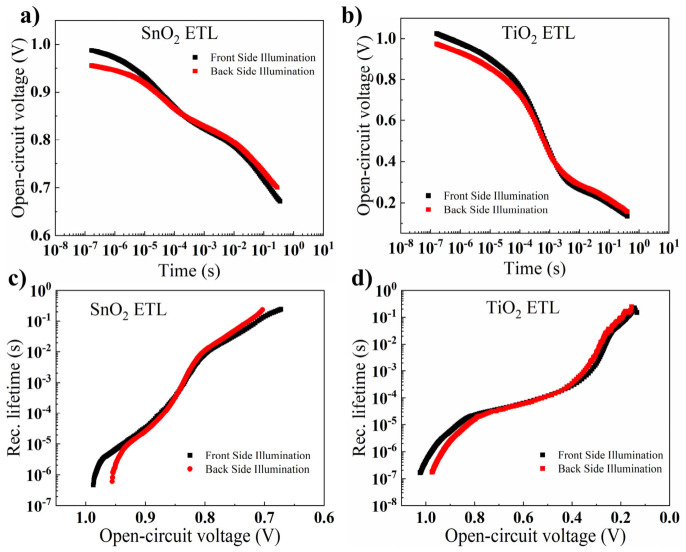
Open-circuit voltage decay curves (**a**,**b**) and the derived recombination carrier lifetimes (**c**,**d**) for the bifacial devices with SnO_2_ and TiO_2_ ETLs, measured under front (black) and back (red) illumination.

Figure 4a,b present the time-resolved *V_oc_* decay profiles for the SnO_2_- and TiO_2_-based devices, respectively, following both front and back illumination. The devices with the SnO_2_ ETL exhibit a remarkably slow and gradual voltage decay. Conversely, the TiO_2_-based devices suffer from a precipitous drop in *V_oc_* almost immediately after the light source is extinguished. The rapid decay in the TiO_2_ architecture indicates a massive depletion of charge carriers driven by severe non-radiative recombination pathways, whereas the prolonged voltage retention in the SnO_2_ devices signifies excellent charge storage capability. Notably, the decay profiles for front and back illumination in the SnO_2_ devices remain closely aligned, underscoring their superior bifaciality.

To quantitatively evaluate these observations, the voltage-dependent recombination lifetimes (*τ_rec_*) were extracted from the OCVD profiles using the relation [49,50]: $\tau_{rec}=-\frac{kT}{q}{\left(\frac{dV_{OC}}{dt}\right)}^{-1}$. The corresponding lifetime versus *V_oc_* plots are shown in Figure 4c (for SnO_2_ ETL) and Figure 4d (for TiO_2_ ETL).

Across the relevant high-voltage operating range (1.0 V to 0.8 V), the SnO_2_-based devices yield significantly longer recombination lifetimes (from ~10^−6^ s to ~10^−2^ s) compared to the TiO_2_-based devices (from ~10^−7^ s to ~10^−5^ s), with the latter showing a hallmark of severe trap-assisted SRH recombination at the ETL interface. These transient results perfectly corroborate our steady-state *V_oc_* vs. light intensity measurements and ideality factor derivations. They provide conclusive temporal evidence that replacing TiO_2_ with SnO_2_ systematically suppresses recombination, thereby maximising carrier lifetimes and enabling the high-efficiency bifacial operation observed in our GA-incorporated perovskite solar cells.

Further, the overall recombination rate and the corresponding transient decay of the *V_oc_* under high-excitation conditions were quantitatively modelled using the following differential equations [51,52,53]:

(1) $$\frac{dn_{oc}}{dt}=\;-\left[k_{bm}n_{oc}^{2}+k_{bulk}n_{oc}+k_{surf}n_{oc}exp\left(\frac{qV_{oc}}{kT}\right)\right]$$

(2) $$\frac{dV_{oc}}{dt}=-\frac{k_{B}T}{q}\;\left[k_{bm}n_{oc}+k_{bulk}+k_{surf}exp\left(\frac{qV_{oc}}{kT}\right)\right]$$

Here, *k_bm_*, *k_bulk_*, and *k_surf_* denote the recombination rate constants for radiative bimolecular recombination, bulk trap-assisted SRH recombination, and surface trap-assisted recombination, respectively. The parameter *n_oc_* represents the density of non-equilibrium, photogenerated charge carriers within the bulk of the perovskite absorber.

To execute a rigorous evaluation of these recombination kinetics across various illumination states, first, we determine *n_oc_* as a function of the transient *V_oc_*. Initially, the steady-state, bias-dependent charge carrier density (*n*) was extracted under AM 1.5G illumination employing standard capacitance-voltage spectroscopy (see Figure S4 in the Supplementary Materials). Following this, the intrinsic carrier concentration (*n_i_*) was derived, enabling the calculation of the *n_oc_* corresponding to each instantaneous *V_oc_* value recorded throughout the decay phase (see Figure S5 in the Supplementary Materials). This extraction and conversion process was performed in accordance with established frameworks and methodologies reported in the literature [54].

**Figure 5:**
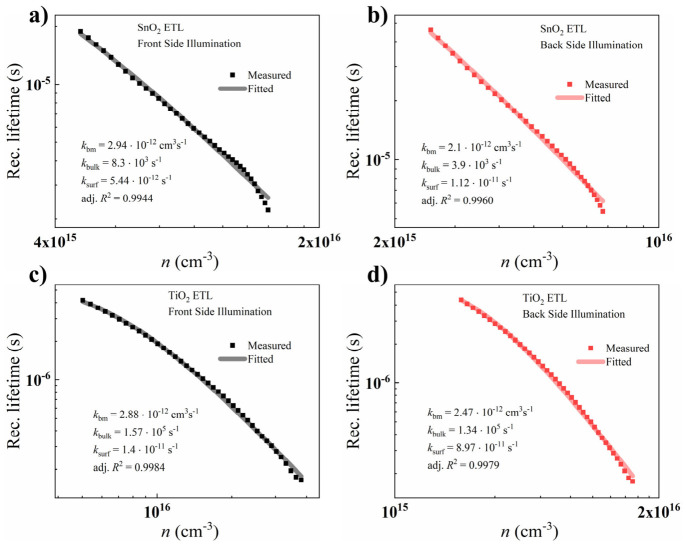
Recombination lifetime as a function of charge carrier density for the bifacial devices utilising (**a**) SnO_2_ under front illumination, (**b**) SnO_2_ under back illumination, (**c**) TiO_2_ under front illumination, and (**d**) TiO_2_ under back illumination. The solid lines represent the theoretical fits based on the differential decay model used to extract the bimolecular, bulk and surface trap-assisted recombination parameters.

Next, the recombination constants (*k_bm_*, *k_bulk_*, and *k_surf_*) were utilised as free fitting parameters to precisely align the theoretical equations with the empirical profiles. The resulting carrier lifetime versus density plots and their corresponding kinetic fits for the devices with the SnO_2_ and TiO_2_ ETLs are presented in Figure 5. The *k_bm_* remain consistent across all device configurations, hovering between 2.1 × 10^−12^ and 2.94 × 10^−12^ cm^3^ s^−1^. Because bimolecular recombination is an intrinsic radiative process governed by the bulk perovskite absorber, this consistency confirms that the fundamental optical quality of the perovskite layer is unaltered by the choice of the underlying ETL, perfectly corroborating our structural and optical findings.

However, a contrast emerges when evaluating the bulk trap-assisted recombination. For the devices employing the TiO_2_ ETL (Figure 5c,d), the fitted *k_bulk_* values are high, reaching 1.57 × 10^5^ s^−1^ and 1.34 × 10^5^ s^−1^ for front and back illumination, respectively. These elevated values signify a high density of non-radiative defect states within the photoactive layer. Conversely, the integration of the SnO_2_ ETL (Figure 5a,b) induces a suppression of these non-radiative losses. The *k_bulk_* values for the SnO_2_-based devices plummet by nearly an order of magnitude, dropping to 8.3 × 10^3^ s^−1^ (front) and 3.9 × 10^3^ s^−1^ (back). This significant reduction suggests that the SnO_2_ underlayer promotes a more favourable crystallisation environment or minimises interface-induced strain, thereby lowering the overall bulk trap density.

Beyond the bulk trap-assisted processes, the *k_surf_* provides a direct quantitative measure of the interfacial defect landscape. Consistent with the trends observed in the bulk, the TiO_2_-based devices exhibited higher *k_surf_* values of 1.4 × 10^−11^ s^−1^ and 8.97 × 10^−11^ s^−1^ under front and back illumination, respectively. These values reflect a severe accumulation of non-radiative recombination centres specifically localised at the TiO_2_/perovskite boundary. In contrast, the SnO_2_-based devices demonstrated a marked suppression, yielding to reduced *k_surf_* values of 5.44 × 10^−12^ s^−1^ and 1.12 × 10^−11^ s^−1^ under front and back illumination, respectively.

Ultimately, this simultaneous reduction in both bulk and surface trap-assisted recombination provides the definitive kinetic mechanism behind the prolonged carrier lifetimes observed in the OCVD measurements. By effectively eliminating the defect states that would otherwise act as severe recombination centres, the optimised SnO_2_ charge extraction interface ensures that photogenerated carriers, even those generated deeper within the bulk during back-side illumination, survive long enough to be collected. This directly yields the enhanced open-circuit voltages and good fill factor under front- and back-side illumination observed in our devices.

To further elucidate the charge carrier dynamics and directly probe the interfacial defect landscape, femtosecond TA spectroscopy was performed. Figure S6 in the Supplementary Information displays the bleach kinetics monitored at 760 nm following excitation with a 440 nm pump pulse. The initial bleach amplitudes were carefully matched by varying the pump pulse energy to ensure comparable photoexcited carrier densities, ruling out variations from higher-order bimolecular recombination [28,29]. Measurements were conducted through both front and back illumination conditions to evaluate the spatial distribution of non-radiative recombination centres selectively. Under both illumination conditions, the perovskite films on TiO_2_ exhibit noticeably faster bleach decay kinetics compared to those on SnO_2_. This accelerated decay is most pronounced under front-side illumination, which selectively probes the region adjacent to the electron transport layer. These TA results provide direct spectroscopic evidence that the devices with TiO_2_ ETL suffer from a significantly higher density of non-radiative trap states. Conversely, the extended carrier lifetimes observed in the SnO_2_-based devices corroborate the suppression of interfacial recombination.

## 4. Conclusions

In summary, we have systematically investigated the influence of the electron transport layer, comparing SnO_2_ and TiO_2_ on the photovoltaic performance and recombination dynamics of bifacial guanidinium-incorporated perovskite solar cells. Devices employing the SnO_2_ ETL demonstrated markedly superior performance under both front- and back-side illumination, culminating in a high bifaciality factor of 0.74 (defined as the ratio of back-to-front power conversion efficiency). Structural and optical characterisations confirmed that while the fundamental bulk crystallinity and light-harvesting capabilities of the perovskite active layer remain unaffected by the underlying substrate, the SnO_2_ ETL provides a distinct optical advantage via enhanced UV-blue transmittance. Beyond these optical benefits, our comprehensive analyses of recombination processes revealed that the primary driver of this enhanced bifacial performance lies in the drastic suppression of non-radiative recombination at the device interface. The SnO_2_ layer effectively mitigated defect states, resulting in a reduction in both bulk and surface trap-assisted recombination rates without disrupting intrinsic bimolecular charge transport. Ultimately, these findings elucidate the critical importance of rational interfacial engineering in maximising the potential of mixed-dimensional perovskite absorbers. By effectively neutralising interfacial defect states and facilitating efficient, balanced charge extraction across varying carrier generation profiles, the SnO_2_ ETL proves to be an indispensable component for the realisation of highly efficient and commercially viable bifacial perovskite optoelectronic devices.

## Figures and Tables

**Table 1:** Summary of photovoltaic parameters for the fabricated bifacial solar cells (8 devices for each ETL).

Device Type	*V_oc_* (V)	*J_sc_* (mA/cm^2^)	*FF*	*PCE* (%)	Bifaciality Factor
ETL SnO_2_ Front Side	1.03 ± 0.01	20.9 ± 1.2	0.64 ± 0.03	13.8 ± 1.3	0.74 ± 0.10
ETL SnO_2_ Back Side	1.01 ± 0.01	14.7 ± 1.3	0.69 ± 0.04	10.2 ± 1.1	
ETL TiO_2_ Front Side	0.99 ± 0.01	16.7 ± 1.1	0.59 ± 0.05	9.7 ± 1.2	0.64 ± 0.12
ETL TiO_2_ Back Side	0.98 ± 0.01	10.9 ± 0.9	0.58 ± 0.04	6.2 ± 1.1	

## Data Availability

The raw data supporting the conclusions of this article will be made available by the authors on request.

## Supplementary Materials

The following supporting information can be downloaded at: https://www.mdpi.com/article/10.3390/ma19112374/s1, Figure S1. Structural and optical characterisation of the active layer. (a) X-ray diffraction pattern of the GA-based quasi-2D perovskite film. (b) Steady-state UV-Vis absorbance spectra of the perovskite films deposited on SnO_2_ (black line) and TiO_2_ (red line) electron transport layers; Figure S2. Optical transmittance spectra of the front electrode substrates; Figure S3. (a) Dark *J-V* characteristics and the extracted built-in voltage, and (b) the corresponding voltage-dependent differential resistance for the SnO_2_- and TiO_2_-based perovskite solar cells; Figure S4. Carrier concentration at a different applied voltage; Figure S5. Calculated *n_oc_* vs. *τ_rec_* in studied PSCs; Figure S6. Transient absorption bleach kinetics (ΔA) probed at 760 nm (440 nm excitation) for guanidinium-incorporated perovskite thin films on TiO_2_ and SnO_2_ under (a) front-side and (b) back-side illumination.

## References

[1] Seo J., Noh J.H., Seok S.I. (2016). Rational Strategies for Efficient Perovskite Solar Cells. Acc. Chem. Res..

[2] Oh K., Jung K., Shin J., Ko S., Lee M.-J. (2021). Novel Intense-Pulsed-Light Synthesis of Amorphous SnO_2_ Electron-Selective Layers for Efficient Planar MAPbI3 Perovskite Solar Cells. J. Mater. Sci. Technol..

[3] Best Research-Cell Efficiency Chart. https://www.nlr.gov/pv/cell-efficiency.

[4] Dong Q., Chen M., Liu Y., Eickemeyer F.T., Zhao W., Dai Z., Yin Y., Jiang C., Feng J., Jin S. (2021). Flexible Perovskite Solar Cells with Simultaneously Improved Efficiency, Operational Stability, and Mechanical Reliability. Joule.

[5] Hu Y., Niu T., Liu Y., Zhou Y., Xia Y., Ran C., Wu Z., Song L., Müller-Buschbaum P., Chen Y. (2021). Flexible Perovskite Solar Cells with High Power-Per-Weight: Progress, Application, and Perspectives. ACS Energy Lett..

[6] He R., Ren S., Chen C., Yi Z., Luo Y., Lai H., Wang W., Zeng G., Hao X., Wang Y. (2021). Wide-Bandgap Organic–Inorganic Hybrid and All-Inorganic Perovskite Solar Cells and Their Application in All-Perovskite Tandem Solar Cells. Energy Environ. Sci..

[7] Parkhomenko H.P., Shalenov E.O., Umatova Z., Dzhumagulova K.N., Jumabekov A.N. (2022). Fabrication of Flexible Quasi-Interdigitated Back-Contact Perovskite Solar Cells. Energies.

[8] Jiang Q., Song Z., Bramante R.C., Ndione P.F., Tirawat R., Berry J.J., Yan Y., Zhu K. (2023). Highly Efficient Bifacial Single-Junction Perovskite Solar Cells. Joule.

[9] Kumar P., Shankar G., Pradhan B. (2022). Recent Progress in Bifacial Perovskite Solar Cells. Appl. Phys. A.

[10] Wang M., Mao P., Zhang P., Lv J., Po-Chuan Y., Li M., Bi W., Wang B., Xing S., Zhong Y. (2026). Lab-to-Fab: Advances and Challenges in Bifacial Perovskite Solar Cells and Modules. Adv. Funct. Mater..

[11] Ou W., Liang J., Guo J., Wang G., Liu Y., Wang Y., Gao Y., Wen J., Li Z., Hong J. (2025). High-Efficiency Fabry-Pérot-Resonance-Based Color-Tunable Bifacial Perovskite Solar Cells for Building Integrated Photovoltaics. Adv. Energy Mater..

[12] Chen X., Yang M., Sun X., Li X., Xie Y., Feng X., Tang J., Yan L., Fan S., Dai S. (2025). Tailoring Thick Film Crystallization and Facet Orientation via Potassium Acetate for Efficient Bifacial Perovskite Solar Cells. ACS Energy Lett..

[13] Nazir G., Rehman A., Hussain S., Aftab S., Patil S.A., Aslam M., Abdel Hafez A.A., Heo K. (2025). Bifacial Perovskite Thin Film Solar Cells: Pioneering the next Frontier in Solar Energy. Nano Energy.

[14] Azmi R., Zhumagali S., Bristow H., Zhang S., Yazmaciyan A., Pininti A.R., Utomo D.S., Subbiah A.S., De Wolf S. (2024). Moisture-Resilient Perovskite Solar Cells for Enhanced Stability. Adv. Mater..

[15] Baumann S., Eperon G.E., Virtuani A., Jeangros Q., Kern D.B., Barrit D., Schall J., Nie W., Oreski G., Khenkin M. (2024). Stability and Reliability of Perovskite Containing Solar Cells and Modules: Degradation Mechanisms and Mitigation Strategies. Energy Environ. Sci..

[16] Hasan S.A.U., Zahid M.A., Park S., Yi J. (2024). Stability Challenges for a Highly Efficient Perovskite/Silicon Tandem Solar Cell: A Review. Sol. RRL.

[17] Dilawar Khan A., Mustajab M., Moeen S., Imran M., Ikram M., Khan Q., Khan M. (2024). Advancements in the Stability, Protection and Lead-Free Strategies of Perovskite Solar Cells: A Critical Review. Environ. Sci. Adv..

[18] Habib H., Rehman S.U., El Hyani H., Sharif M.N., Tan F., Wang K.-F. (2025). Degradation Pathways in Perovskite Solar Cells: Strategies for Enhancing Stability. Energy Technol..

[19] Alotaibi M.H., Alzahrani Y.A., Arora N., Alyamani A., Albadri A., Albrithen H., Al-Lehyani I.H., Alenzi S.M., Alanzi A.Z., Alghamdi F.S. (2020). Halide Versus Nonhalide Salts: The Effects of Guanidinium Salts on the Structural, Morphological, and Photovoltaic Performances of Perovskite Solar Cells. Sol. RRL.

[20] Guvenc C.M., Toso S., Ivanov Y.P., Saleh G., Balci S., Divitini G., Manna L. (2025). Breaking the Boundaries of the Goldschmidt Tolerance Factor with Ethylammonium Lead Iodide Perovskite Nanocrystals. ACS Nano.

[21] Zhang Y., Park N.-G. (2022). Quasi-Two-Dimensional Perovskite Solar Cells with Efficiency Exceeding 22%. ACS Energy Lett..

[22] Shi X., Ding Y., Zhou S., Zhang B., Cai M., Yao J., Hu L., Wu J., Dai S., Nazeeruddin M.K. (2019). Enhanced Interfacial Binding and Electron Extraction Using Boron-Doped TiO_2_ for Highly Efficient Hysteresis-Free Perovskite Solar Cells. Adv. Sci..

[23] Giordano F., Abate A., Correa Baena J.P., Saliba M., Matsui T., Im S.H., Zakeeruddin S.M., Nazeeruddin M.K., Hagfeldt A., Graetzel M. (2016). Enhanced Electronic Properties in Mesoporous TiO_2_ via Lithium Doping for High-Efficiency Perovskite Solar Cells. Nat. Commun..

[24] Ranjan S., Ranjan R., Tyagi A., Rana K.S., Soni A., Kodali H.K., Dalal V., Singh A., Garg A., Nalwa K.S. (2022). Low-Temperature Microwave Processed TiO_2_ as an Electron Transport Layer for Enhanced Performance and Atmospheric Stability in Planar Perovskite Solar Cells. ACS Appl. Energy Mater..

[25] Mohd Firdaus F.F., Ludin N.A., Umar A.A., Syafiq U., Goje A.A., Tasmia T.A. (2026). Low Temperature Tin Oxide Electron Transport Layers for Flexible Perovskite Solar Cells: A Review. Sol. Energy.

[26] Uddin A., Yi H. (2022). Progress and Challenges of SnO_2_ Electron Transport Layer for Perovskite Solar Cells: A Critical Review. Sol. RRL.

[27] Lu Y., Shih M.-C., Tan S., Grotevent M.J., Wang L., Zhu H., Zhang R., Lee J.-H., Lee J.-W., Bulović V. (2023). Rational Design of a Chemical Bath Deposition Based Tin Oxide Electron-Transport Layer for Perovskite Photovoltaics. Adv. Mater..

[28] Pydzińska-Białek K., Szeremeta J., Wojciechowski K., Ziółek M. (2019). Insights into the Femtosecond to Nanosecond Charge Carrier Kinetics in Perovskite Materials for Solar Cells. J. Phys. Chem. C.

[29] Szulc K., Pydzińska-Białek K., Ziółek M. (2023). Modeling of Charge Injection, Recombination, and Diffusion in Complete Perovskite Solar Cells on Short Time Scales. Materials.

[30] Yerlanuly Y., Shalenov E.O., Parkhomenko H.P., Kiani M.S., Kukhayeva Z., Ng A., Jumabekov A.N. (2024). Elucidating the Hysteresis Effect in Printed Flexible Perovskite Solar Cells with SnO_2_ Quantum Dot- and PCBM-Based Electron Transport Layers. Heliyon.

[31] Solovan M.M., Mostovyi A.I., Aidarkhanov D., Parkhomenko H.P., Akhtanova G., Schopp N., Asare E.A., Nauruzbayev D., Kaikanov M., Ng A. (2023). Extreme Radiation Resistance of Self-Powered High-Performance Cs0.04Rb0.04(FA0.65MA0.35)0.92Pb(I0.85Br0.14Cl0.01)3 Perovskite Photodiodes. Adv. Opt. Mater..

[32] Azamat A.K., Parkhomenko H.P., Kiani M.S., Ng A., Jumabekov A.N. (2024). Self-Powered Printed Flexible Bifacial Perovskite Photodetector. ACS Appl. Opt. Mater..

[33] Sahare S., Solovan M., Smirnova M., Scheibe B., Jancelewicz M., Nowaczyk G., Kempiński M., Ziółek M. (2024). MXenes as a Hole Transport Interfacial Layer for Efficient and Air-Stable Quasi-2D Perovskite Solar Cells. J. Mater. Chem. C.

[34] Sze S.M., Li Y., Ng K.K. (2021). Physics of Semiconductor Devices.

[35] Parkhomenko H.P., Solovan M.N., Mostovyi A.I., Ulyanytsky K.S., Maryanchuk P.D. (2017). Temperature Dependences of the Electrical Parameters of Anisotype NiO/CdTe Heterojunctions. Semiconductors.

[36] Würfel P. (2005). Physics of Solar Cells: From Principles to New Concepts.

[37] Green M.A. (1998). Solar Cells: Operating Principles, Technology and System Applications.

[38] Brus V.V., Schopp N., Ko S.-J., Vollbrecht J., Lee J., Karki A., Bazan G.C., Nguyen T.-Q. (2021). Temperature and Light Modulated Open-Circuit Voltage in Nonfullerene Organic Solar Cells with Different Effective Bandgaps. Adv. Energy Mater..

[39] Wu W.Q., Yang Z., Rudd P.N., Shao Y., Dai X., Wei H., Zhao J., Fang Y., Wang Q., Liu Y. (2019). Bilateral Alkylamine for Suppressing Charge Recombination and Improving Stability in Blade-Coated Perovskite Solar Cells. Sci. Adv..

[40] Akhtanova G., Parkhomenko H.P., Vollbrecht J., Mostovyi A.I., Schopp N., Brus V. (2025). Surface Recombination in Organic Solar Cells: Intrinsic vs. Doped Active Layer. Org. Electron..

[41] Akhtanova G., Parkhomenko H.P., Asanov N., Mostovyi A.I., Schopp N., Kaikanov M., Brus V.V. (2025). Organic Solar Cells for Space Applications: The Crucial Role of Active Layer Thickness. Adv. Opt. Mater..

[42] Hartnagel P., Kirchartz T. (2020). Understanding the Light-Intensity Dependence of the Short-Circuit Current of Organic Solar Cells. Adv. Theory Simul..

[43] Liu Z., Niu S., Wang N. (2017). Light Illumination Intensity Dependence of Current-Voltage Characteristics in Polymer Solar Cells with Solution-Processed Titanium Chelate as Electron Extraction Layer. Sol. Energy.

[44] Parkhomenko H.P., Yerlanuly Y., Brus V.V., Jumabekov A.N. (2024). Effect of Mild Mechanical Stresses on Device Physics of Slot-Die Coated Flexible Perovskite Solar Cells. Org. Electron..

[45] Zeiske S., Li W., Meredith P., Armin A., Sandberg O.J. (2022). Light Intensity Dependence of the Photocurrent in Organic Photovoltaic Devices. Cell Rep. Phys. Sci..

[46] Wang Y., Wu B., Wu Z., Lan Z., Li Y., Zhang M., Zhu F. (2017). Origin of Efficient Inverted Nonfullerene Organic Solar Cells: Enhancement of Charge Extraction and Suppression of Bimolecular Recombination Enabled by Augmented Internal Electric Field. J. Phys. Chem. Lett..

[47] Brus V.V., Lang F., Bundesmann J., Seidel S., Denker A., Rech B., Landi G., Neitzert H.C., Rappich J., Nickel N.H. (2017). Defect Dynamics in Proton Irradiated CH3NH3PbI3 Perovskite Solar Cells. Adv. Electron. Mater..

[48] Zaban A., Greenshtein M., Bisquert J. (2003). Determination of the Electron Lifetime in Nanocrystalline Dye Solar Cells by Open-Circuit Voltage Decay Measurements. ChemPhysChem.

[49] Fahrenbruch A., Bube R. (2012). Fundamentals of Solar Cells: Photovoltaic Solar Energy Conversion.

[50] Pockett A., Eperon G.E., Peltola T., Snaith H.J., Walker A., Peter L.M., Cameron P.J. (2015). Characterization of Planar Lead Halide Perovskite Solar Cells by Impedance Spectroscopy, Open-Circuit Photovoltage Decay, and Intensity-Modulated Photovoltage/Photocurrent Spectroscopy. J. Phys. Chem. C.

[51] Vollbrecht J., Brus V.V. (2020). On the Recombination Order of Surface Recombination under Open Circuit Conditions. Org. Electron..

[52] Vollbrecht J., Brus V.V., Ko S.-J., Lee J., Karki A., Cao D.X., Cho K., Bazan G.C., Nguyen T.-Q. (2019). Quantifying the Nongeminate Recombination Dynamics in Nonfullerene Bulk Heterojunction Organic Solar Cells. Adv. Energy Mater..

[53] Vollbrecht J., Brus V.V. (2021). Effects of Recombination Order on Open-Circuit Voltage Decay Measurements of Organic and Perovskite Solar Cells. Energies.

[54] Parkhomenko H.P., Solovan M.M., Sahare S., Mostovyi A.I., Aidarkhanov D., Schopp N., Kovaliuk T., Kaikanov M., Ng A., Brus V.V. (2024). Impact of a Short-Pulse High-Intense Proton Irradiation on High-Performance Perovskite Solar Cells. Adv. Funct. Mater..

