# Supplementary material for: Unravelling Recombination Processes in Bifacial Guanidinium-Incorporated Perovskite Solar Cells with SnO2 and TiO2 ETLs
Source: Materials (Basel). 2026 Jun 3;19(11):2374. doi: 10.3390/ma19112374 (PMC13257555; doi:10.3390/ma19112374)
Supplement: Supplementary file 1 [file materials-19-02374-s001.zip › materials-4340666-supplementary.pdf]

Supplementary Materials

Unravelling Recombination Processes in Bifacial Guanidinium-Incorporated Perovskite Solar Cells with SnO_2 and TiO_2 ETLs

Hryhorii Parkhomenko, Adem Karakuzu, Sanjay Sahare, Mykhailo Solovan * and Marcin Ziółek *

Faculty of Physics and Astronomy, Adam Mickiewicz University, Uniwersytetu Poznańskiego 2, 61-614 Poznań, Poland

* Correspondence: mykhailo.solovan@amu.edu.pl or myksol@st.amu.edu.pl (M.S.); marziol@amu.edu.pl (M.Z.)

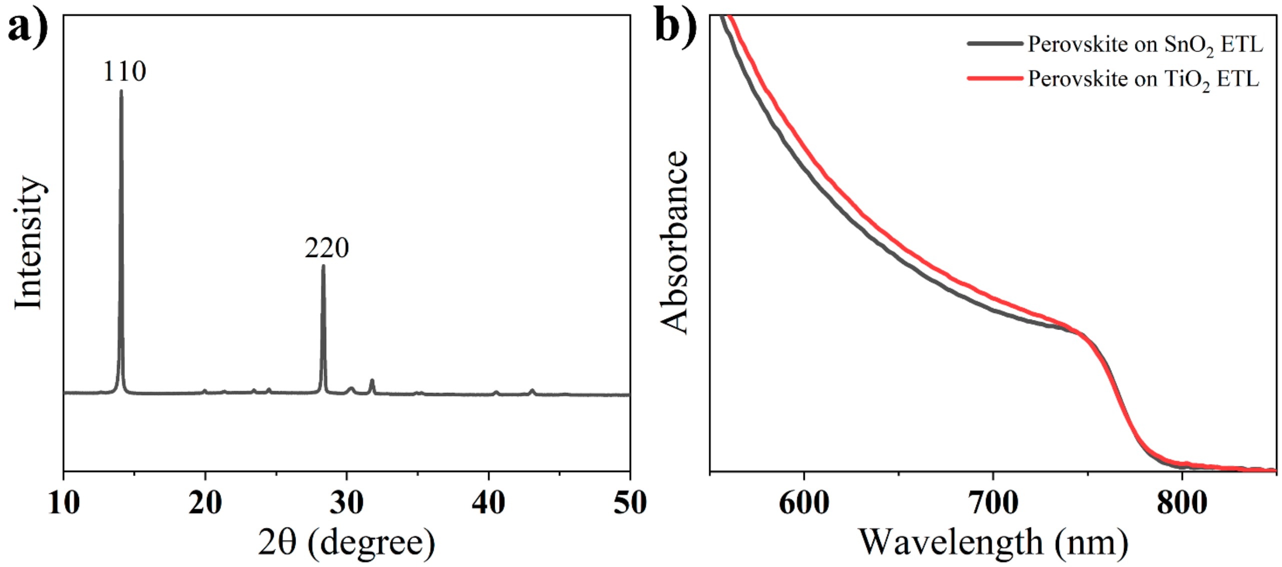

Figure S1. Structural and optical characterisation of the active layer. **(a)** X-ray diffraction pattern of the GA-based quasi-2D perovskite film. (b) Steady-state UV-Vis absorbance spectra of the perovskite films deposited on SnO_2 (black line) and TiO_2 (red line) electron transport layers.

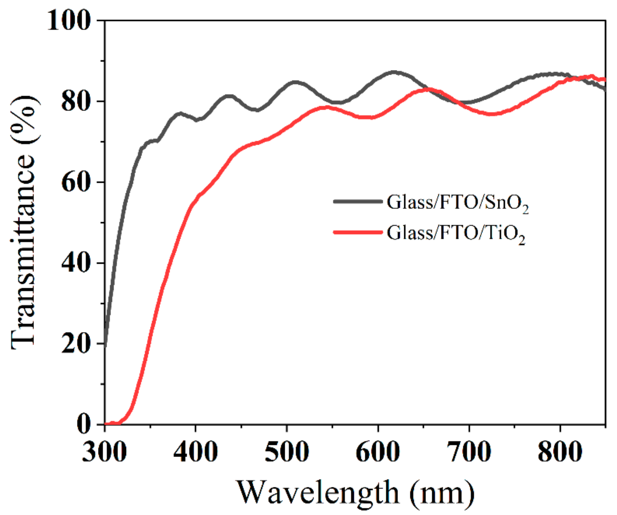

Figure S2. Optical transmittance spectra of the front electrode substrates.

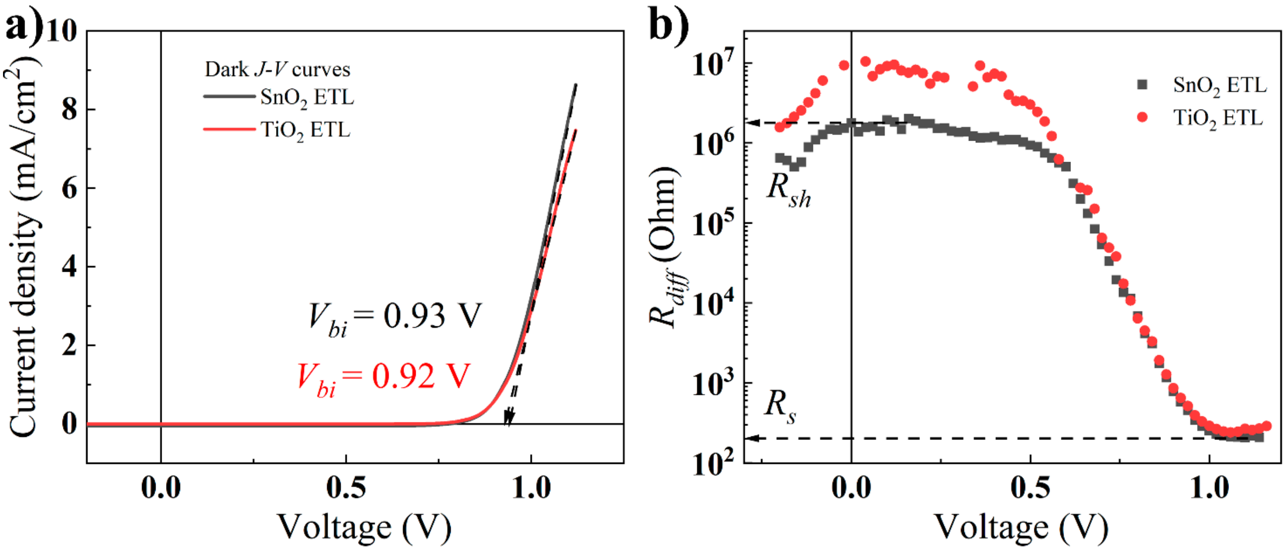

Figure S3. (a) Dark *J-V* characteristics and the extracted built-in voltage, and **(b)** the corresponding voltage-dependent differential resistance for the SnO_2- and TiO_2-based perovskite solar cells.

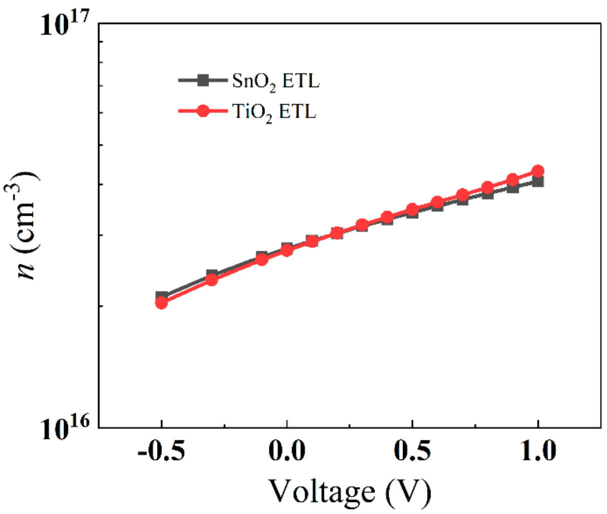

Figure S4. Carrier concentration at a different applied voltage.

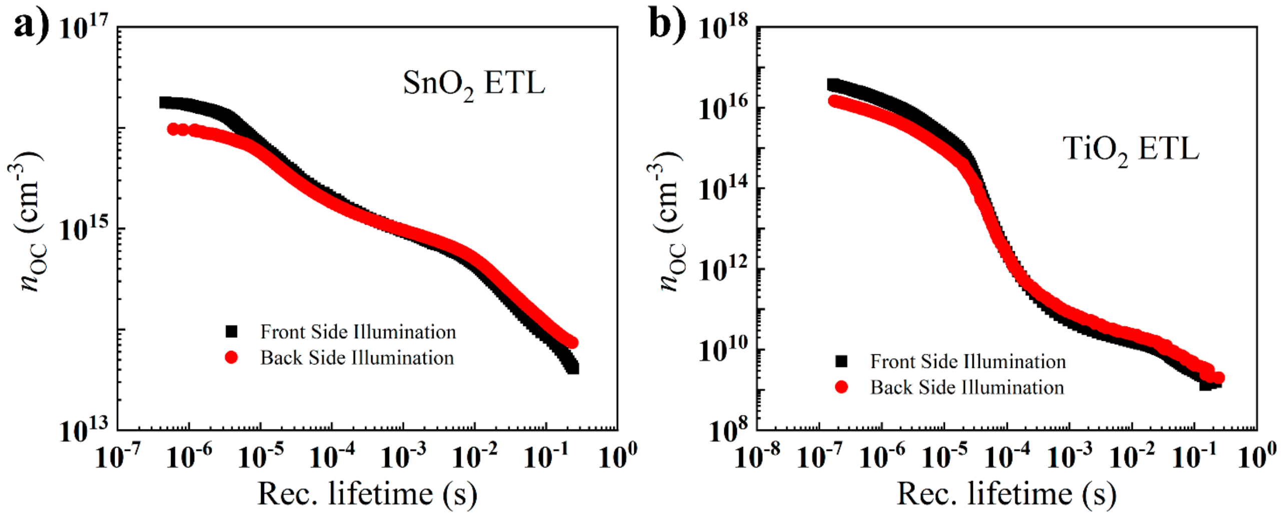

Figure S5. Calculated n_{oc} vs. τ_{rec} in studied PSCs.

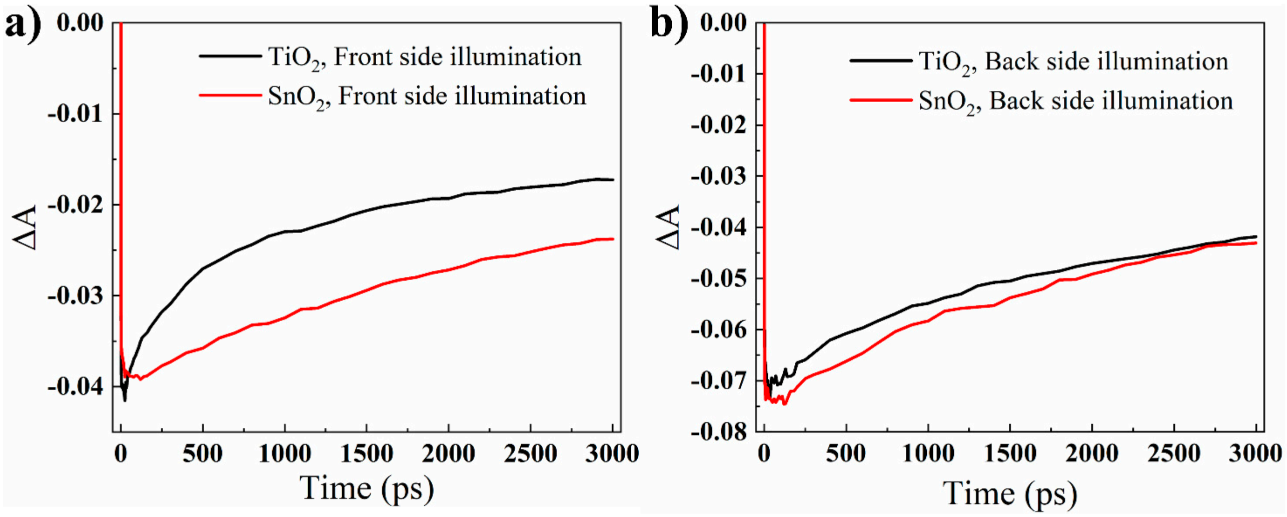

Figure S6. Transient absorption bleach kinetics (ΔA) probed at 760 nm (440 nm excitation) for guanidinium-incorporated perovskite thin films on TiO_2 and SnO_2 under **(a)** front-side and **(b)** back-side illumination.
